# Tune Deafness: Processing Melodic Errors Outside of Conscious Awareness as Reflected by Components of the Auditory ERP

**DOI:** 10.1371/journal.pone.0002349

**Published:** 2008-06-11

**Authors:** Allen Braun, Joe McArdle, Jennifer Jones, Vladimir Nechaev, Christopher Zalewski, Carmen Brewer, Dennis Drayna

**Affiliations:** 1 Language Section, National Institute on Deafness and Other Communication Disorders, National Institutes of Health, Bethesda, Maryland, United States of America; 2 Section on Systems Biology of Communication Disorders, National Institute on Deafness and Other Communication Disorders, National Institutes of Health, Bethesda, Maryland, United States of America; 3 Audiology Unit, National Institute on Deafness and Other Communication Disorders, National Institutes of Health, Bethesda, Maryland, United States of America; The Rockefeller University, United States of America

## Abstract

Tune deafness (TD) is a central auditory processing disorder characterized by the inability to discriminate pitch, reproduce melodies or to recognize deviations in melodic structure, in spite of normal hearing. The cause of the disorder is unknown. To identify a pathophysiological marker, we ascertained a group of severely affected TD patients using the Distorted Tunes Test, an ecologically valid task with a longstanding history, and used electrophysiological methods to characterize the brain's responses to correct and incorrect melodic sequences. As expected, we identified a neural correlate of patients' unawareness of melodic distortions: deviant notes modulated long-latency auditory evoked potentials and elicited a mismatch negativity in controls but not in affected subjects. However a robust P300 was elicited by deviant notes, suggesting that, as in blindsight, TD subjects process stimuli that they cannot consciously perceive. Given the high heritability of TD, these patients may make it possible to use genetic methods to study cellular and molecular mechanisms underlying conscious awareness.

## Introduction

The appreciation of music requires that the brain process and decode a complex stream of acoustic signals in order to extract and consciously perceive salient features such as pitch, harmony, and melody. If this is done successfully, the qualities of music that are consciously experienced can evoke a wide range of emotions, memories, or images [1]. Understanding how this process fails in disorders of musical perception may thus provide insight into a wide range of normal cognitive functions.

Tune deafness (TD) is one such disorder [2], [3] **.** Individuals with this well-recognized phenotype are unable to accurately perceive pitch or reproduce melodies or to recognize deviations in melodic structure, despite the fact that they perform within normal limits on tests of peripheral hearing.

The TD phenotype is assigned using the Distorted Tunes Test (DTT), a convenient, ecologically valid, and reliable instrument that has been in use for more than four decades [2], [3]. Although TD is distinct syndrome in that the phenotype is explicitly defined by reproducibly poor performance on the DTT, it may be related to a disorder termed congenital amusia, which is defined using different criteria [4], [5]. (**TEXT S1**)

TD is a common disorder, affecting approximately 2% of the population [3] and is also highly heritable [2], [6]. Yet the physiological defects at the core of the disorder are unknown. Once identified, these mechanisms should be of value in identifying the genetic variants that underlie this condition, which could potentially provide insight into its molecular and cellular basis.

In an effort to identify a pathophysiological marker in TD, we ascertained a group of severely affected subjects. We then used electrophysiological methods (electroencephalography and event related potentials, EEG/ERP) to characterize the brain's responses to a series of familiar melodies that contained correct and deviant terminal notes. This paradigm, a modification of the DTT, allowed us to directly investigate the central, clinically relevant features of the disorder–TD subjects' inability to recognize melodic deviations (see Methods).

EEG/ERP methods are ideal for such studies because they offer excellent temporal resolution and provide quantitative information about both perception and higher order processing of acoustic information. These methods have been widely used for years to study music processing in the human brain [1], [7]. ERP components, evoked responses to acoustic stimuli occurring at characteristic latencies, are well described and have been associated with specific functions. For example, cortical components of the auditory evoked response (designated P1, N1, and P2) provide precise information about the initial processing of acoustic stimuli in primary auditory cortex and early auditory association areas [8].

There are also distinct, well-described ERP components that are signatures of the brain's response to deviant (mismatched, “oddball” or otherwise distorted) auditory stimuli. These include the so-called mismatch negativity (MMN) [9] and the P300 [10]. While their physiological and cognitive features differ, both can be generated in response to unexpected or deviant pitch changes, or to violations of rules that govern pattern and sequencing of auditory information P300: [11]; MMN [12].

Since by definition, tune deaf subjects are unaware of deviations in melodic structure, we predicted that both the MMN and the P300 response to such deviations would be absent in these subjects. We have found that this is not the case. While our results provide a potential electrophysiological substrate for auditory unawareness, they also suggest that tune deaf subjects are processing musical abnormalities, but are doing so outside of conscious awareness.

## Results

**Figure 1:**
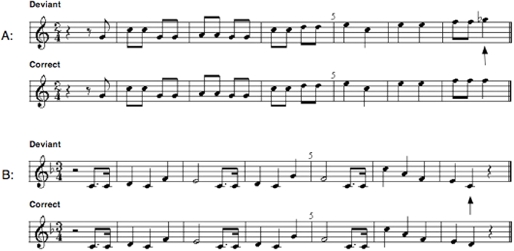
Examples of correct and incorrect melodies. (A) Bingo and (B) Happy Birthday are illustrated with correct versions on the bottom and incorrect versions at the top. Deviant terminal notes are indicated by arrows.

EEG data were acquired while subjects heard different versions of familiar tunes that either contained a correct (standard) or an aberrant (deviant) note at the end of the melodic sequence (Figure 1) (see Methods). They were instructed to listen but not specifically asked to detect abnormal notes, permitting evaluation of subjects' natural responses to melodic deviations, without the superimposition of a vigilance task.

### Long Latency Auditory Evoked Potentials

**Figure 2:**
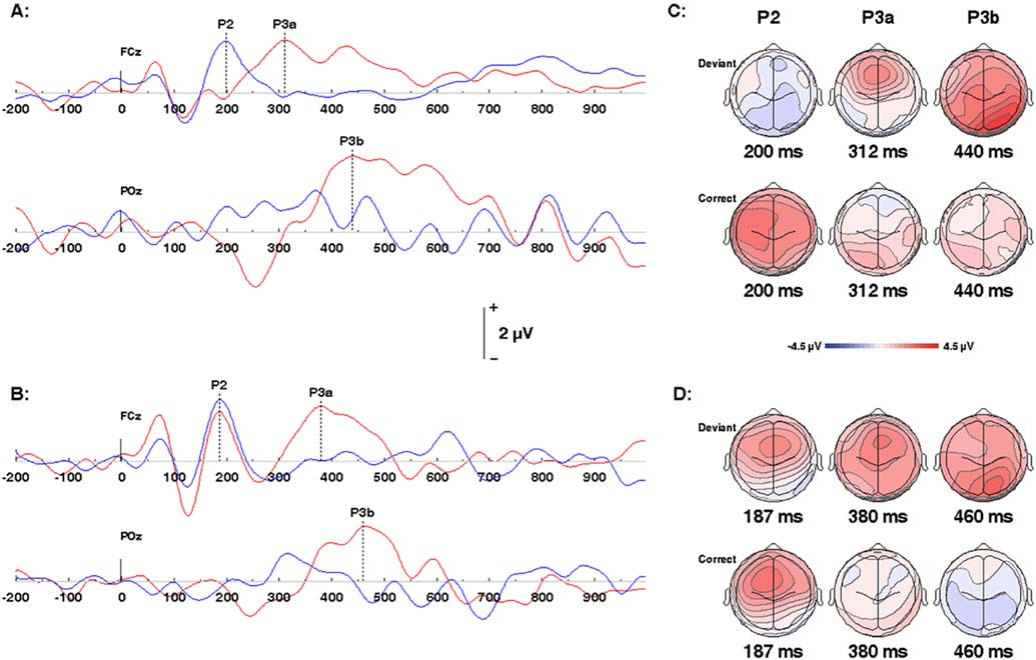
ERPs elicited by the correct and incorrect melodies' final notes. (A) Grand averaged ERP waveforms from the control group at electrode coordinates FCz and POz from correct notes (indicated in blue) and deviant notes (red). (B) ERPs from the same electrode coordinates and conditions from the TD group. For both groups, correct notes elicited a clear P2 component. Following deviant notes however, the P2 was strongly diminished for the control group but showed no significant attenuation for TD participants. A 2-way ANOVA revealed an interaction of Group x Note: F(1,16) = 5.6, p = .0309; and main effects of Note: F(1,16) = 7.99, p = .012; and Group: F(1,16) = 5.55, p = .0315. Planned comparisons indicated a significant difference between correct and deviant notes for controls (correct 2.62 µV, deviant 0.66 µV, t (16) = 3.72, p = .001) but not TD (correct 3.16 µV, deviant 2.99 µV, NS). In contrast, deviant notes elicited components in the P300 latency range for both control and TD participants. Two-way ANOVA showed a significant main effect of Note for P3a (F(1,16) = 13.59, p = .002), but no effect, of Group or Group x Note interaction. Planned comparisons revealed that the P3a was significantly greater in response to deviant than correct notes in both groups (controls, deviant 3.97 µV, correct 1.02 µV, p = .0069; TD, deviant 2.50 µV, correct 1.00 µV, p = .0149). Two-way ANOVA showed a significant main effect of note for P3b (Note: F(1,16) = 8.73, p = .009), an effect of group (F(1,16) = 10.61, p = .005) but no group x note interaction. Planned comparisons showed that the P3b was significantly greater in response to deviant than correct notes in both groups (controls, deviant 4.43 µV, correct 2.02 µV, p = .0293; TD, deviant 2.54 µV, correct 0.99 µV, p = .0192). (C) Topographic maps of the P2, P3a, and P3b ERP distributions from deviant and correct final notes and mean group latencies for each component. (D) Corresponding topographic maps from the TD group.

Characteristic long latency auditory evoked potentials (AEPs) containing clear P1, N1 and P2 components were elicited in response to standard tones in both TD and control subjects. These components displayed typical latencies and waveform morphology (Fig 2A, C; **Movie S1** **,** **S2**). In contrast, the P2 component was markedly attenuated for melodic abnormalities in controls but not TD subjects. (Fig 2A, C; see also **Movie S1** **,** **S2**).

### Mismatch Negativity

**Figure 3:**
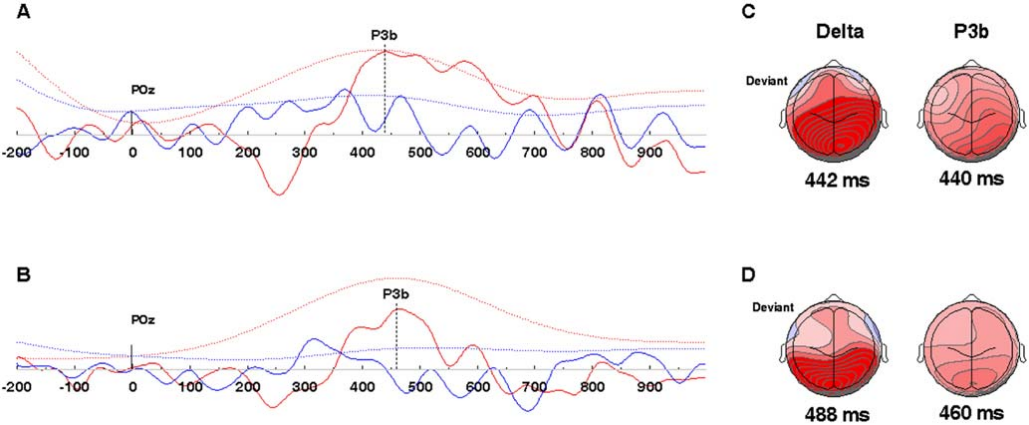
Grand averaged difference waveforms (deviant-standard) illustrating the MMN at electrode coordinate FCz. (A) Depicts the MMN waveform component (left) and topographic distribution (right) from the control group. (B) Depicts the comparable waveform and topographic map from TD participants. A 1-way ANOVA indicated a significant main effect of group (F(1,16) = 5.46, p = .0328).

The later components of the AEP, including P2, may be modulated when a MMN is elicited at the same latencies by deviant auditory stimuli. Consistent with this, a MMN with characteristic morphology and a latency overlapping that of the P2 was evoked by the abnormal melodic sequences in controls, but not in TD subjects (Fig 3). These features may thus provide an index of the TD subjects' failure to explicitly process the deviant tones.

### P300

In contrast, a typical P300 response was evoked by deviant notes in both controls and TD subjects. This included P3a and P3b components, both showing characteristic latencies and waveform morphology. The P3a was more robust in anterior channels; the waveform from channel FCz is illustrated in Fig 2A. P3b was greater in posterior channels; the waveform from channel POz is illustrated in Fig 2B (see also **Movie S1** **,** **S2**). There was a trend toward prolonged P300 latencies, particularly for the P3a, in TD subjects (Figure 2A), although these differences were not statistically significant. Overall, results suggest that although late responses to melodic abnormalities may be delayed in TD, subjects are clearly processing these abnormalities.

**Figure 4:**
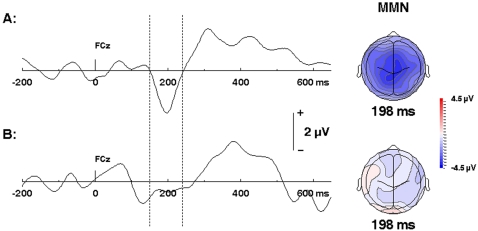
(A and B) Grand averaged ERP waveforms (solid lines) and evoked delta power (dashed lines) compared at electrode coordinate POz. Waveforms in blue (both ERP and evoked power) represent responses to correct notes, while waveforms in red represent responses to deviant notes. (A) Comparison for controls and (B) tune-deaf participants. Both groups produced a peak evoked delta response for deviant tunes with peak latencies approximating those of the P3b. Peak delta responses were markedly lower for correct notes. A 2-way ANOVA showed a significant main effect of note for evoked delta power (Note: F(1,16) = 12.33, p = .0029; controls, deviant 12.52 µV, correct 5.85 µV; TD, deviant 13.47 µV, correct 3.25 µV), but no group effect, and no group x note interaction. (C) Topographic maps of the evoked delta response (left) and the P3b component (right) for controls. (D) Comparable maps for TD participants. Note: In this figure, evoked delta waveforms and topographic maps have been scaled for display purposes so that they can be directly compared to the ERP data.

Beyond their characteristic latencies and waveform morphologies, components of the P300 response have been specifically linked in human subjects with evoked oscillations in the delta band [13]. To evaluate these relationships in our subjects, we compared delta oscillations evoked by standard and deviant tones (see Methods). Both controls and TD subjects showed a characteristic association between the P300 and delta; a significant increase in delta power was evoked by deviant but not standard tones, overlapping principally with the P3b response (Figure 4).

## Discussion

Tune deafness has been characterized in a number of ways. However, the pathophysiological mechanisms that underlie the defining feature of the disorder–subjects' inability to recognize distortions in melodic structure–have rarely been investigated in context. Here we have attempted to do so in a group of severely affected TD subjects. We used a modification of the Distorted Tunes Test, a clinically relevant, ecologically valid paradigm–that is, one that makes it possible to investigate, in context, the essential behavioral features of this disorder.

We used EEG/ERP to measure the brain's responses to correct and distorted melodies. (**Text S1**). Two of these responses, the MMN and P300, are established indices of change detection and, since TD subjects are unaware of melodic distortions, we predicted that both of these responses would be absent in these individuals in response to distorted notes. This was not the case. The pattern of responses we detected was more complex, suggesting that this disorder may not only provide insights into music processing, but also into brain mechanisms that underlie both conscious and unconscious perception.

The long-latency components of the AEP reflect the earliest cortical responses to auditory stimuli. Of these, the P1 and N1 components were evoked by deviant as well as standard notes–that is, they were unmodulated by melodic abnormalities–in controls and TD subjects. Significant group differences were instead related to the later, P2 component, which was selectively attenuated in response to deviant tones in controls. This was not unexpected. Nittono and coworkers, using a similar paradigm, reported an attenuation of the P2 component in response melodic abnormalities in normal individuals [14]. Such a response was absent in TD subjects. (Figure 1A, B, **Movie S1** **,** **S2**).

Because the role of the P2 is less well understood than that of the other long-latency AEP components, it is not clear what its selective attenuation may denote in physiological terms. However, the P2 may be obscured when a MMN, typically generated within same time window, is present, and this is the difference between TD and controls that may be most critical.

The MMN, is a well-established marker of change detection, most commonly elicited by deviant auditory stimuli [9] A so-called pattern MMN, generated in response to deviations in complex auditory patterns or learned sequences[15], can be readily evoked by musical pattern deviations [12].

Such a MMN, with a characteristic latency and morphology, was elicited by melodic abnormalities in controls (Figure 2), likely accounting for attenuation of the P2 component. No MMN was generated by deviant stimuli in TD subjects. Normal AEPs, unmodulated by a MMN, indicate that these subjects perceive each note, but are simply unable to detect melodic errors.

In stark contrast, a robust P300 was evoked by melodic abnormalities in both TD subjects and normal controls. The P300 is another well-established signature of change detection; it is frequently differentiated from MMN in that while it can be generated automatically [16], it is more readily modulated by attention (**Text S1**).

Although the P300 response was originally described as single entity, it is now clear that it consists of two distinct components - P3a and b [10]. Both of these, each characterized by typical latencies and waveform morphologies, were evoked in TD subjects as well as controls (Figure 1A, B, **Movie S1** **,** **S2**).

Our results therefore represent an apparent paradox. The absence of the MMN provides the predicted marker for auditory unawareness that is the hallmark of tune deafness. But the unexpected presence of the P300 indicates that TD subjects are at the same time processing the very abnormalities that they cannot consciously perceive. A recent paper, demonstrating a dissociation between conscious perception and behavioral performance in this patient population [17] provides support for this notion.

Such a phenomenon, sometimes referred to as knowledge without awareness, has been recognized for some time [18] in conditions such as blindsight [19], prosopagnosia [20] or deaf-hearing [21]. However in these disorders the symptoms–paradoxical responses to sensory stimuli that are not consciously perceived–are associated with structural brain lesions (although subliminal awareness may also be demonstrated in normal subjects by manipulating stimuli under experimental conditions [22]).

In TD, the symptoms may be related to anatomical distinctions between the MMN and P300. Although both the MMN and P300 are indices of change detection, they are structurally dissociable: the neural assemblies that generate these waveforms are situated in different regions of the brain. The sources of the MMN are located for the most part in unimodal auditory areas of the superior temporal gyrus [9] while the sources of the P300 are located in heteromodal regions of the frontoparietal cortex. These heteromodal regions are downstream projection areas which normally receive information that has already been processed in unimodal sensory cortices[23]. (**TEXT S1**).

These anatomical differences suggest a model that may account for knowledge without awareness in tune deafness. Since it is the MMN that distinguishes TD and controls, our results suggest that conscious perception in TD subjects is likely disrupted at the level of the unimodal auditory cortex. This supports a more general notion that neural computations in early auditory areas are necessary for determining whether deviant auditory information is consciously perceived. Previous studies have indeed suggested that feature-specific neurons within these auditory regions regulate the access to auditory awareness in a bottom up fashion, and that responses underlying the MMN itself may operate as a gateway to consciousness[24].

At the same time, the robust generation of the P300 suggests that deviant stimuli are nonetheless being discriminated and selectively processed in higher order frontoparietal cortices, having bypassed earlier mechanisms that regulate conscious perception.

A plausible explanation for this might be that acoustic stimuli are reaching the cortical sources of the MMN and P300 via independent, parallel pathways. This in turn, suggests a pathophysiological mechanism for TD similar to the one that accounts for the symptoms of blindsight,. In blindsight, perceptual processes mediated by direct projections from thalamus to primary visual cortex are disturbed, while alternate pathways to association areas remain intact [19], [25]. Similar parallel projections have been demonstrated in the auditory system [26], where direct projections from the thalamus to primary areas in the auditory core and belt are complemented by parallel, independent pathways to association areas in the parabelt and adjacent heteromodal cortices. In TD, as in blindsight, it may be the direct route in which processing is abnormal, while transfer of afferent information through alternate pathways is preserved.

Independent activation of the different cortical areas is clearly not the rule in the normal brain. The natural activation pattern may be consistent with a model [18] in which perceptual awareness is part of a serial process connecting two modules–with heteromodal systems in the frontoparietal cortices receiving input that has already been extensively processed in unimodal sensory areas upstream. In this model, the modules could become uncoupled in disorders such as tune deafness or blindsight, where they would operate independently. In such cases, information would reach the heteromodal cortices, but in a fashion that cannot support conscious awareness.

In light of this, TD may provide a novel opportunity to study conscious perception. Unlike lesion-based disorders such as blindsight it should be possible to study the process in a brain that is structurally intact, and in a patient population that is far larger. In addition, TD is a highly heritable condition, and investigations currently underway may identify the genetic variants that underlie this condition. The tune deaf population may thus constitute a group in which the problem of consciousness might be approached at the cellular and molecular level using the tools of genetic research.

## Methods

### Participants

Tune deaf subjects were ascertained by random screening of 1218 individuals in two metropolitan areas. Among those scoring in the lowest 10^th^ percentile on the DTT, 20 subjects were identified who had normal hearing, were medically and neurologically normal and were free of other confounding factors. Of these, eight subjects consented to participate in this protocol; one subject was excluded due to the presence of excessive EEG artifact, so that seven TD subjects (4 females, 3 males, ages 18–33, 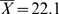, SD = 4.64 years) were ultimately included**.** Ten healthy control subjects who performed within normal limits on the DTT were also studied (2 females, eight males, ages 22–56, 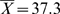, SD = 9.98 years).

All participants were right-handed [27] native English speakers, with normal or corrected vision. All were free of neurological or medical illness, and were not taking any psychoactive medications at the time of their participation in the study. All subjects had normal hearing bilaterally (audiologic evaluations for speech and pure tones were performed in 6 TD subjects; one was assessed using the Five Minute Hearing Test) [28]. All participants provided written informed consent after the nature and possible consequences of the studies were explained to them, in accordance with protocols 00-DC-0176 and 02-DC-0178, approved by the NINDS/NIDCD Institutional Review Board.

### Materials

The EEG paradigm employed a modification of the DTT [3] in which 102 familiar, western tonal melodies (mean of 23 notes, range 12–34) were used. Each melody was processed, using Mozart software (version 3.2), so that one version contained the correct (standard) note and a second version contained a single aberrant (deviant) tone at end of the melodic sequence. Deviant end notes were generated by varying the pitch of final notes between 1 and 3 semitones (17 to 83 Hz). Melodies were produced in pure tones with Mozart software in MIDI format and then converted to WAV format.

### Stimulus presentation

Participants were seated facing a 34 cm LCD monitor and were asked to fixate on a 1.5 cm cross at a 5 degree visual angle from 1.5 m and listen to a series of melodies. Melodies were presented at 90 Db through a single speaker, located 1.5 m in front of the participant. The interstimulus interval between melodies was 3s. Of the melodies prepared, 180 (87 correct, 93 containing deviant notes) were presented in random order, using Neuroscan STIM software [29]. Subjects, were instructed to listen but not specifically asked to detect abnormal notes, permitting evaluation of subjects' natural responses to melodic deviations, without the superimposition of a vigilance task.

### EEG recording

All electrophysiological signals were recorded using 9 mm sintered silver silver-chloride electrodes. EEG was recorded from a 60 channel electrode cap, conforming to the extended 10–20 electrode placement system [29] and referenced to linked ears. Bipolar leads were placed above and below the left eye, in order to measure the electro-oculogram (EOG). Electrical impedance between the ground electrode and all mono and bipolar electrodes was maintained below 5 KΩ. Data were digitized at 500 points per second and recorded continuously between 1.0 and with 100 Hz using two 32-channel Synamp bio-amplifiers. All EEG data were recorded in an electrostatically shielded chamber.

### Data analysis

Individual EEG trials were visually inspected and those that contained artifacts or exceeded 100µV of EOG were excluded from the analysis. ERP averaging was time-locked to the onset of the final notes in the remaining trials. Waveform peak amplitudes and latencies were derived from a 1000 ms ERP with a 200 ms baseline interval. For the P1, N1, P2, N2, P3a, and P3b peak amplitudes and latencies were derived for both correct and deviant terminal notes. The P1 peak was operationally defined as the maximum positive amplitude between 30 and 110 ms at electrode FCz, the N2 as the maximum negative amplitude between 70 and 160 ms at FCz, the P2 as the maximum positive amplitude between 100 and 260 ms at FCz, the N2 as the maximum negative amplitude between 200 and 350 ms at electrode POz, the P3a as the maximum positive amplitude between 270ms and 500 ms at electrode FCz, and the P3b as the maximum positive amplitude between 350 and 500 ms at electrode POz. In order to obtain peak amplitudes and latencies for the MMN, ERP waveforms from deviant notes were subtracted from those of correct notes. The MMN peak was defined as the maximum negative amplitude between 150 and 250 ms at electrode FCz.

Separate 2-way repeated measures ANOVA were conducted in order to determine the effects of note type (correct or deviant) and group (control or TD) on the amplitudes and latencies of the P1, N1, P2, N2, P3a, and P3b. Due to potential covariation between experimental conditions, introduced by the repeated measures design, the Huynh and Feldt Epsilon correction was applied to each calculated F-statistic. All tests were held to a family-wise α of .05. Hypotheses specific mean comparisons were performed using paired t-tests, with Bonferroni corrections in order to maintain the specified experiment-wise type I error rate.

Induced delta power was calculated using the Event-Related Bandpower function of Neuroscan's Edit software [29].In order to calculate evoked power in the delta frequency range, complex demodulation was applied to individual EEG epochs from 200 ms prior to 1000 ms post terminal note using a 1.5 Hz central frequency and 1.5 Hz half-band width (48 Db rolloff). Average power and variance were then computed across EEG epochs on the resulting complex time series. The delta peak was operationally defined as the maximum power within the time series.

## Supporting Information

Text S1 Supplementary Notes.
(0.07 MB DOC)

Movie S1 Correct Note. Comparison of ERP responses to correct notes from the control and tune-deaf groups. Electrophysiological findings are represented topographically on 3D head models: Left (control) and right (tune-deaf) panels also display the ERP waveform as recorded from electrode coordinate FCz as a frame of reference, timers, and color scales.
(5.83 MB MOV)

Movie S2 Deviant Note. Comparison of ERP responses to deviant notes from the control and tune-deaf groups. Electrophysiological findings are represented topographically on 3D head models: Left (control) and right (tune-deaf) panels also display the ERP waveform as recorded from electrode coordinate FCz as a frame of reference, timers, and color scales.
(6.24 MB MOV)
