# Supplementary material for: Tune Deafness: Processing Melodic Errors Outside of Conscious Awareness as Reflected by Components of the Auditory ERP
Source: PLoS One. 2008 Jun 11;3(6):e2349. doi: 10.1371/journal.pone.0002349 (PMC2396485; doi:10.1371/journal.pone.0002349)
Supplement: Text S1 — Supplementary Notes. (0.07 MB DOC) [file pone.0002349.s001.doc]

Tune deafness: Processing melodic errors outside of conscious awareness as reflected by components of the auditory ERP

Braun, A.^1^ *, McArdle, J.^1^, Jones, J^2^., Nechaev, V.^1^, Zalewski, C.^3^, Brewer, C.^3^, Drayna, D. ^2^

^1^ Language Section, ^2^ Section on Systems Biology of Communication Disorders and ^3^ Audiology Unit, National Institute on Deafness and Other Communication Disorders, National Institutes of Health, Bethesda, MD 20892

**Supplementary Notes:**

**Tune deafness and congenital amusia:** The symptoms of tune deafness have been recognized for many years, as a form of apperceptive auditory agnosia, a canonical central auditory processing disorder[1].

Since the 1940s, large scale studies have ascertained members of this population using the Distorted Tunes Test (DTT)[2] – an instrument designed to measure a core deficit of this disorder – subjects’ inability to recognize deviations in melodic structure. Since that time, the DTT has been repeatedly validated and used to establish the prevalence of this phenotype in a number of different groups, which together comprise over 4000 individuals

Recently, a comparable phenotype has been defined using a standardized psychoacoustic test battery (the Montreal Battery of Evaluation of Amusa). Using this instrument, symptomatic individuals have been shown to have deficits in fine grained pitch discrimination, pitch contour and interval recognition, as well as rhythm discrimination, all presenting in the context of normal hearing and normal auditory memory [3,4].

We have also evaluated measures of central auditory processing in a group of TD subjects ascertained using the DTT[5]. These studies have revealed a number of similarities with features described in congental amusia, but due to differences in ascertainment and details of the auditory tests employed, it is unclear at this point whether or not the phenotypes described by our two groups are identical. It will be important in the future to use both the MBAE and the DTT and associated test batteries in the same cohort of subjects in order to establish this.

Estimates of prevalence also differ between the two groups. In the cohorts identified using the MBAE, prevalence is estimated at approximately 4% [6] (consistent with original estimates using the DTT). Our own estimate derives from a random sampling of 1218 subjects. The distribution of scores on the DTT was continuous in this population, however subjects scoring below the10th percentile have clear and reproducible symptoms. After removing individuals with impaired hearing and other confounding factors, our experience suggest that individuals affected specifically with TD constitute approximately 2% of the general population.

**Electrophysiological studies in amusia:** There have been no previous studies evaluating electrophysiological responses to melodic deviations in tune deaf subjects. However, EEG/ERP methods have been used to estimate responses to infrequent pitch deviant tones (designed to elicit an MMN) or in auditory classification tasks (designed to elicit the P300) in patients with symptoms of amusia following stroke. Both the MMN[7] and P300 [8] were attenuated in these brain damaged individuals.

A single study has investigated electrophysiological responses to acoustic stimuli in patients with congenital amusia (not secondary to stroke) [9]. This study used a formal oddball paradigm, in which subjects monitored monotonic tone sequences for pitch changes and indicated the presence of deviant targets when these were detected. The authors reported abnormal right-lateralized N2-P3 responses to large pitch changes in amusic subjects.

**MMN, P300, attention and consciousness:** While both the MMN and the P300 are indices of change detection, they have each been associated with different cognitive or neuropsychological features. One of the principal differences between these responses is their susceptibility to modulation by attention[10].

The MMN is frequently characterized as preattentional or automatic [10] and it can be elicited in the absence of vigilance, without overt attention paid to target stimuli. On the other hand, in the typical P300 paradigm, a higher amplitude response is generally evoked when subjects are monitoring a stimulus train in order to detect a target. While there are differences between the P300a and b in this context (the P3a response may be more automatic, P3b more readily task-modulated) both components are modulated by attention**.**

Attentional modulation of the evoked responses may not be relevant in the present study, in which subjects were not instructed to monitor the melodic sequences to detect the presence of deviant stimuli. If we had expected, but not seen, a P300 under these circumstances, the absence of the response might well have represented a confound secondary to these task features. Since we observed a P300 response, even though the chances of detecting this may have been minimized in the absence of vigilance, this indicates that the response represents a clear reaction to deviant stimuli, even in the absence of attention (and, as we argue in these subjects, in the absence of conscious awareness). It will be interesting to see if the P300 response we observed can indeed be modulated by attention in characteristic ways.

Because of their contrasting relationships to attention, it has sometimes been assumed that the MMN and P300 are similarly related to conscious perception. Thus, the preattentional MMN might operate outside of conscious awareness, while the P300 reflects conscious processing of deviant stimuli. This interpretation likely inaccurate; while attention is closely associated to consciousness, they are not equivalent.

In fact, the MMN is exquisitely sensitive to normal physiological fluctuations in consciousness[11] it disappears during coma, and has been used as a tool to detect minimally preserved consciousness and effectively predict recovery [12]. On the other hand, the P300 may actually persist under these conditions, [13] suggesting that the P300, while it is modulated by attention, does not reflect conscious perception. Indeed, it has been argued that the P300 represents an automatic and unconscious change detection mechanism [14,15].

**Sources of the AEP, MMN and P300:** Sources of the long-latency components of the AEP are thought to be multi-focal, generated in unimodal auditory areas. The generators of the MMN may differ from those of the AEP [10] although some studies have suggested that the MMN may actually represent delayed, stimulus-specific alteration of AEP components themselves[16]. Nevertheless, it is clear that the sources of the MMN are located principally in the unimodal auditory cortex[17]; although secondary sources may also include projections areas in the mid-ventrolateral prefrontal cortex[18].

A consistent body of evidence suggests that sources of the P3a are located lateral prefrontal areas, while the P3b may have multiple sources, including the inferior parietal lobe in the region of the supramarginal gyrus, and the superior and posterior parietal (and adjacent cingulate) cortex[19].

References

1. Griffiths TD (2002) Central auditory pathologies. Br Med Bull 63: 107-120.

2. Kalmus H, Fry DB (1980) On tune deafness (dysmelodia): frequency, development, genetics and musical background. Ann Hum Genet 43: 369-382.

3. Foxton JM, Dean JL, Gee R, Peretz I, Griffiths TD (2004) Characterization of deficits in pitch perception underlying 'tone deafness'. Brain 127: 801-810.

4. Peretz I, Ayotte J, Zatorre RJ, Mehler J, Ahad P, Penhune VB, Jutras B (2002) Congenital amusia: a disorder of fine-grained pitch discrimination. Neuron 33: 185-191.

5. Jones J, Brewer C, Zalewski C, Parker S, Drayna D (2005) Auditory & Phonological Processing Abilities in Tune Deaf Subjects.

6. Sloboda JA, Wise KJ, Peretz I (2005) Quantifying tone deafness in the general population. Ann N Y Acad Sci 1060: 255-261.

7. Kohlmetz C, Altenmuller E, Schuppert M, Wieringa BM, Munte TF (2001) Deficit in automatic sound-change detection may underlie some music perception deficits after acute hemispheric stroke. Neuropsychologia 39: 1121-1124.

8. Munte TF, Schuppert M, Johannes S, Wieringa BM, Kohlmetz C, Altenmuller E (1998) Brain potentials in patients with music perception deficits: evidence for an early locus. Neurosci Lett 256: 85-88.

9. Peretz I, Brattico E, Tervaniemi M (2005) Abnormal electrical brain responses to pitch in congenital amusia. Ann Neurol 58: 478-482.

10. Naatanen R, Paavilainen P, Rinne T, Alho K (2007) The mismatch negativity (MMN) in basic research of central auditory processing: A review. Clin Neurophysiol 118: 2544-2590.

11. Sabri M, De Lugt DR, Campbell KB (2000) The mismatch negativity to frequency deviants during the transition from wakefulness to sleep. Can J Exp Psychol 54: 230-242.

12. Wijnen VJ, van Boxtel GJ, Eilander HJ, de GB (2007) Mismatch negativity predicts recovery from the vegetative state. Clin Neurophysiol 118: 597-605.

13. Laureys S, Perrin F, Faymonville ME, Schnakers C, Boly M, Bartsch V, Majerus S, Moonen G, Maquet P (2004) Cerebral processing in the minimally conscious state. Neurology 63: 916-918.

14. Johnson R, Jr. (1988) Scalp-recorded P300 activity in patients following unilateral temporal lobectomy. Brain 111 ( Pt 6): 1517-1529.

15. Sommer W, Leuthold H, Matt J (1998) The expectancies that govern the P300 amplitude are mostly automatic and unconscious. Behavioral and Brain Sciences 21: 149-150.

16. Jaaskelainen IP, Ahveninen J, Bonmassar G, Dale AM, Ilmoniemi RJ, Levanen S, Lin FH, May P, Melcher J, Stufflebeam S, Tiitinen H, Belliveau JW (2004) Human posterior auditory cortex gates novel sounds to consciousness. Proc Natl Acad Sci U S A 101: 6809-6814.

17. Picton TW, Alain C, Otten L, Ritter W, Achim A (2000) Mismatch negativity: different water in the same river. Audiol Neurootol 5: 111-139.

18. Rinne T, Alho K, Ilmoniemi RJ, Virtanen J, Naatanen R (2000) Separate time behaviors of the temporal and frontal mismatch negativity sources. Neuroimage 12: 14-19.

19. Linden DE (2005) The p300: where in the brain is it produced and what does it tell us? Neuroscientist 11: 563-576.
